# Comparative Transcriptome and Proteome Analysis Provides New Insights Into the Mechanism of Protein Synthesis in Kenaf (*Hibiscus cannabinus* L.) Leaves

**DOI:** 10.3389/fpls.2022.879874

**Published:** 2022-06-21

**Authors:** Chao Zhang, Yong Deng, Gaoyang Zhang, Jianjun Li, Aiping Xiao, Lining Zhao, Anguo Chen, Huijuan Tang, Li Chang, Gen Pan, Yingbao Wu, Jiangjiang Zhang, Cuiping Zhang, Ziggiju Mesenbet Birhanie, Hui Li, Juan Wu, Dawei Yang, Defang Li, Siqi Huang

**Affiliations:** ^1^Institute of Bast Fiber Crops, Chinese Academy of Agricultural Sciences, Changsha, China; ^2^School of Life Sciences, Shangrao Normal University, Shangrao, China

**Keywords:** kenaf, transcriptome, gene expression, proteome, protein synthesis

## Abstract

Given the rising domestic demand and increasing global prices of corn and soybean, China is looking for alternatives for these imports to produce animal fodder. Kenaf (*Hibiscus cannabinus* L.) has great potential as a new forage source, due to abundant proteins, phenols and flavonoids in its leaves. However, few studies have evaluated the mechanism of protein synthesis in kenaf leaves. In the current work, compared with kenaf material “L332,” the percentage of crude protein content in leaves of material “Q303” increased by 6.13%; combined with transcriptome and proteome data, the kenaf samples were systematically studied to obtain mRNA-protein correlation. Then, the genes/proteins related to protein synthesis in the kenaf leaves were obtained. Moreover, this work detected mRNA expression of 20 differentially expressed genes (DEGs). Meanwhile, 20 differentially expressed proteins (DEPs) related to protein synthesis were performed parallel reaction monitoring. Fructose-1,6-bisphosphatase (*FBP*), nitrite reductase (*NirA*), prolyl tRNA synthase (*PARS*) and glycine dehydrogenase (*GLDC*) presented increased mRNA and protein levels within kenaf leaves with high protein content. Based on the obtained findings, *FBP*, *NirA*, *PARS*, and *GLDC* genes may exert a vital function in the protein synthesis of kenaf leaves. The results provide a new idea for further studying the potential genes affecting the quality trait of protein content in kenaf leaves and provide gene resources and a theoretical foundation for further cultivating high protein kenaf varieties.

## Introduction

The impact of global climate change on agricultural production has been a hotspot for academia at home and abroad; the large impact of artificial pollutants on the natural environment plays a leading role in reducing crop productivity and is the real cause of different abiotic stress states (Akcura et al., 2019; Bilen et al., 2019; Tang et al., 2019; Kashif et al., 2020a,b; Li et al., 2021). In China, soybean and corn imports exceeded 110 million tons in 2020, and demand for feed grains is still increasing. Additionally, the rise in international food prices has diverted the intent of feed producers to look for alternatives to reduce or replace feed grains. Hence, owing to the abundant proteins, phenols, and flavonoids in its leaves, kenaf has great potential as a new forage source. However, few studies have evaluated the molecular mechanisms of protein synthesis in kenaf leaves. Kenaf (*Hibiscus cannabinus* L.), a kind of annual bast fiber crop of Hibiscus in Malvaceae family, represents the heliophilous crop that grows in tropical and temperate zones (Cheng et al., 2004). The kenaf plant can provide 2 fiber types, phloem and core. Bast fiber locates within phloem part in the kenaf stems, which is suitable for textile and industrial purposes, such as in paper, rope, textiles, and carpets (Falasca et al., 2014). The core part is located inside the stem of kenaf, which is rich in cellulose and hemicellulose and can be used as an absorbent material. For example, it can be utilized in indoor acoustic panels, thermal insulation panels, indoor sound insulation boards and heat insulation boards (Okuda and Sato, 2004). Phloem and core can be applied alone or concurrently in producing biological composites or biofuels (Saba et al., 2015). The chemical components of the volatile oil from kenaf leaves were analyzed (Pascoal et al., 2015). A total of 58 components of the volatile oil were identified. Volatile oil has phytotoxicity and antifungal activity. Kenaf have the potential to deal with a wide range of heavy metal pollution with high economic benefits (Deng et al., 2017). Its economic value is reflected not only in fiber utilization but also in important research prospects in the materials, bioenergy, medicine, feed, papermaking and carbon sink trade (Ayadi et al., 2017). In addition, in production practice, kenaf has the characteristics of fast growth and large biomass and can be used as livestock feed. Kenaf has fast growth speed, contains flavonoids and polyphenols, exhibits strong insect resistance, and does not need to apply insecticides, which are natural green protein feeds (Swingle et al., 1978; Birhanie et al., 2021). With the rapid development of animal husbandry and breeding in China, the feed industry has also emerged. High-quality protein feed has become an urgent necessity, especially in southern China. Due to the humid and hot climate and other reasons, there is a lack of high-quality and high-yield plant protein feed. Therefore, carrying out kenaf feed research, breeding kenaf feed varieties and strengthening the research on kenaf feed nutritional value will bring economic and ecological benefits.

Plant protein accumulation is a complex process accompanied by a large amount of protein synthesis and the transcription of many genes (Shen et al., 2019; Xie et al., 2019). The results showed that the crude protein content of different genotypes was significantly different, indicating that genetic factors exerts an essential function in the protein content of soybean (Wang et al., 2021). Studies on genes related to protein synthesis have been carried out in many species, including *Arabidopsis thaliana*, cassava, wheat, radish, phaseolus and soybeans. These studies show that sucrose phosphate synthase (SPS), fructose-1,6-bisphosphatase (FBP), nitrite reductase (NiR), fructose-1,6-bisphosphatase aldolase (ALDO), prolyl tRNA synthase (PARs), and glycine dehydrogenase (GLDC) (Jeannin et al., 1976; Okamura-Ikeda et al., 1993; Sahrawy et al., 2004; Wu et al., 2015; Lv et al., 2017; Huang et al., 2020) were related to protein synthesis. The *A. thaliana NRT* gene regulates NO_3_^–^ absorption and plant dynamic responses to changes in nitrogen content in the environment (Wang et al., 2012). Additionally, Tiwari et al. (2019) found that the post-translational regulation of the *NR* gene highly influences the content of free amino acids and nitrate. Therefore, further genetic analyses on protein synthesis is of necessity for protein accumulation.

Although numerous researches have been reported on the impacts of abiotic stress or cutting methods on the protein content of kenaf leaves, little progress has been made on the molecular mechanism of protein synthesis in kenaf leaves. Most of the 15 reports on kenaf transcriptome sequencing were the study of differentially expressed genes after biological stress (Niu et al., 2015), and there are no reports on kenaf leaf protein synthesis. The understanding of the molecular characteristics of protein synthesis in kenaf leaves is limited, making it difficult to select and breed kenaf varieties with high protein content in leaves. The recent progress in sequencing methods like transcriptome and proteome techniques, brings us a great convenience in the measurement of gene expression and protein abundance. The sequencing methods have turned into a powerful instrument for discovering novel genes and improving the protein content of kenaf leaves (Jérme and Richard, 2008).

Therefore, the objective of this research was to examine the transcriptome and proteome of two kenaf material: “L332” and “Q303,” which assistant by high-throughput sequencing technology. We also assessed their difference in the percentage of crude protein content in leaves. Furthermore, we explored the potential genes affecting the quality trait of protein content. The comprehensively analysis strengthen our understanding of kenaf protein synthesis at the molecular level.

## Materials and Methods

### Plant Growth and Sampling

Kenaf seed materials “Q303” and “L332,” which are cultivated in southern China, were provided by the Institute of Bast Fiber Crops, Chinese Academy of Agriculture Sciences. In this study, it was observed that the leaves of kenaf material “L332” belong to the lobed-leaf type and the leaves of kenaf material “Q303” belong to the round-like leaf type. Our experimental site was located in the Bairuopu Innovative Experimental Base at the Institute of Bast Fiber Crops, Chinese Academy of Agricultural Sciences in Changsha. The experimental area had a typical subtropical continental monsoon climate. A field with the same water and fertilizer conditions was selected as the experimental site. Two kenaf materials were sown on May 16, 2019 and harvested on October 20, 2019. The whole growth period lasted for 155 days. At the mature stage (September 17), all leaves within 50 cm below the top of the kenaf plant were collected and mixed for carrying out further analyses. The mixed samples were from the leaves of 20 kenaf plants. This work set 3 biological replicates for every kenaf material. For every kenaf material, this study gathered 30 g leaf samples from an individual plant. We froze sample leaves with liquid nitrogen at once, followed by preservation under −80°C until the application for RNA isolation, protein separation, and quantitative real-time PCR analyses.

### Morphological Character Assays and Extraction of Total Protein of Kenaf Leaves

This study determined kenaf leaf size using the graduated scale, while leaf weight using the electronic balance, and the total protein of kenaf leaves using the Kjeldahl method (Grzeszczuk et al., 2018). The leaves of kenaf were subjected to 0.5-h deactivation under 105°C, followed by drying under 65°C to constant weight. Subsequently, the leaves of kenaf were crushed to determine the protein content. We prepared 3 biological replicates of every kenaf material.

### RNA Extraction and cDNA Library Construction

The leaves in the biological replicate for every sample were grinded to powder with liquid nitrogen. According to Chai et al. (2014), extraction of total RNA in two kenaf leaf samples was conducted. Total RNA extracted from kenaf leaves was dissolved in RNase-free water (TIANGEN, China). The A260/A280 ratio was measured using the Nanodrop ND-1000 system (Thermo Scientific, United States) and adopted for checking the RNA concentration in kenaf leaves, and 1.5% agarose gels were adopted for detecting the integrity of the RNA. After the RNA of kenaf samples was qualified, the library was prepared for the qualified samples. cDNA libraries were created with the use of the NEBNext^®^ Ultra™ RNA Library Prep Kit for Illumina^®^ (NEB, United States) in accordance with specific protocols. Kenaf mRNA purification was conducted using the extracted total RNA (4 μg) with oligo (dT) magnetic beads (Thermo Scientific) following the instructions of the manufacturer. Then, synthesis of first- and second-strand cDNA by reverse transcription was conducted, with mRNA being a template. After purification of double-stranded cDNA and then end repair, polyadenylation, and addition of adaptor sequences, DNA fragment sorting and PCR amplification were performed. Finally, a cDNA library was constructed. After purification of PCR products (AMPure XP system), we assessed cDNA library quality on an Agilent Bioanalyzer 2100 system. Finally, the cDNA library was constructed and sequenced after quality inspection.

### RNA Sequencing and Transcriptome Assembly

The cDNA libraries for those 3 biological replicates of kenaf samples were constructed according to Cui et al. (2019). Subsequently, sequencing was carried out by an Illumina NovaSeq 6000 instrument by Shanghai Applied Protein Technology Co., Ltd. Sequencing was completed by an Illumina NovaSeq 6000 instrument from Shanghai Applied Protein Technology Co., Ltd. (Shanghai, China). Besides, through CASAVA base calling analysis, raw imaging data acquired by Illumina high-throughput sequencing were transformed into original sequenced reads. Besides, these findings were kept as the fastq file format. In the fastq file, four lines are regarded as a basic unit and correspond to the sequencing information of a sequence. The clean data (clean reads) were obtained by filter_fq software through filtering out low-quality and adaptor sequences as well as reads that contained the poly-N sequences. High-quality sequence data can be obtained by filtering out sequences that are too short or contain uncertain bases. We calculated the Q20, Q30, GC levels, as well as clean reads sequence duplicate degrees. Using Trinity software package^1^ (Grabherr et al., 2011) to assemble and splice the clean reads, data assembly was later conducted. Trinity software workflow was used for *de novo* transcriptome assembly. It contains 3 separate software modules, respectively, Inchworm, Butterfly and Chrysalis. In addition, RNA-seq read data were processed by the three software modules in turn. Transcripts <200 bp were fully removed.

### Bioinformatics Analyses

This work annotated *de novo* assembled unigenes with the National Center for Biotechnology Information (NCBI) non-redundant protein sequence database (NR), the database for manual annotation and protein sequence reviewing (Swiss-Prot), a protein family database (Pfam), an integrated database on the basis of Gene Ontology (GO), as well as a genome database on Kyoto Encyclopedia of Genes and Genomes (KEGG), and threshold value was set as an *E*-value < 10^–5^. After gene GO annotation, the annotated genes were categorized in accordance with GO (Biological processes BPs, Cellular Components CCs, Molecular Functions MFs). KEGG database contributes to studying genes and expression information as a whole network. KEGG pathway analysis was accomplished through KOBAS 2.0 test statistical enrichment (Chen et al., 2011).

In this experiment, the Fragments Per Kilobase of exon model per Million mapped fragments (FPKM) value was used for transcription and quantification of gene expression, and DEG-seq was applied to explore the differential expression of samples. We controlled false discovery rate using the *p*-value adjustment methods of Benjamini and Hochberg (1995). The screening criteria for obvious differences were a *p* < 0.05 and | log2 (FC)| ≥ 1. The expression was deemed to be different between the two samples. Cluster Profiler R software was employed to realize GO as well as KEGG analysis on DEGs. Ap, the PlantTFDB^2^ was used to analyze transcription factors (TFs) in DEGs.

### Protein Separation

Protein was extracted in kenaf leaves of every sample based on previous description, with three biological replicates for each treatment (An et al., 2018). BCA Protein Assay Kit (Bio-Rad, United States) was adopted for determining the protein concentration.

### Protein Digestion and TMT Labeling

Protein was digested based on Liu et al.’s method (Liu et al., 2018), with some modifications. In filter-aided sample preparation (FASP Digestion), we poured all samples in the buffer [consisting of 150 mM Tris-HCl (pH 8.0), 4% SDS, 100 mM DTT], followed by 7-min boiling of mixed solution and cooling immediately. The samples were treated following specific protocols of TMT Kit (Thermo Scientific, United States). This work thereafter utilized TMT 6-plex to categorize trypsin (that contained 100 μg protein) for 126-tag (HPC-1), 127-tag (HPC-2), 128-tag (HPC-3), 129-tag (LPC-1) 130-tag (LPC-2) and 131-tag (LPC-3) labeling. The label assimilation was checked, and all the above 6 labeled samples in diverse set were later combined.

### HPLC Fractionation and LC–MS/MS Analysis

Thereafter, this work loaded the mixed peptide sample to the reversed-phase trap column (nanoViper C18, 100 μm*2 cm; Thermo Scientific Acclaim PepMap100) equipped with the C18 reversed-phase analytical column (length, 10-cm; 3-μm resin; inner diameter, 75-μm; Thermo Scientific Easy Column) within buffer A consisting of 0.1% formic acid, followed by separation using the gradient buffer B (0.1% formic acid and 84% acetonitrile) at a 300 nl/min flow rate under the control by IntelliFlow Technology. Afterward, peptides were detected using Q-Exactive High-Resolution Mass Spectrometer (Thermo Scientific). This study obtained survey scans at the 70,000 at m/z 200 resolution, whereas HCD spectra were obtained at 17,500 and 35,000 at m/z 200 (TMT 6plex and 10plex, respectively), with isolation width being set at 2 m/z. Meanwhile, this work set the underfill ratio (specifying the minimal target value percentage possibly reached at the maximal fill time) and normalized collision energy as 0.1% and 30 eV separately.

### Sequence Database Search and Data Analysis

This work obtained original MS data in RAW files. This work utilized Proteome Discoverer 1.4 software as well as MASCOT engine (Matrix Science, London, United Kingdom; version 2.2) for searching MS/MS spectra against kenaf database with the settings below, enzyme trypsin and two as the maximal missed cleavage allowed; variable oxidation modification (M), fixed carbamidomethyl modification (C), TMT-6plex (K) and TMT-6plex (N-term); and mass tolerance for peptide ions and fragment ions were 20 ppa and 0.1 Da, separately, at both protein and peptide levels of false discovery rate (FDR) < 0.01. Proteins were detected and quantified using specific protein peptides. Differentially abundant proteins (DAPs) were detected by the thresholds of FC ≤ 0.83 or ≥ 1.2 and *p* < 0.05.

The GO annotation based on three categories, including BPs, MFs, and CCs, together with KEGG analysis, was carried out by Fisher’s exact test (*p* < 0.05). This work also adopted STRING 9.0 software for constructing the PPI network^3^. Later, Java Treeview software^4^ and Cluster 3.0^5^ were utilized for hierarchical clustering.

### Quantitative Real-Time PCR

By adopting SYBR Green PCR master mix (Aidlab Biotechnologies, Co., Ltd.), qRT-PCR was conducted to analyze 20 potential DEGs levels according to the manufacturers’ instructions on Bio-Rad CFX96 Touch detection system (Bio-Rad, Richmond, CA, United States). The total RNA isolation method and cDNA preparation method were the same as previous description. Genomic DNA was removed using gDNA Eraser (Tsingke), later, the isolated total RNA was prepared into cDNA with Goldenstar™ RT6 cDNA Synthesis Kit Ver. 2 through reverse transcription. Primers were designed with Primer 5.0 software (Supplementary Table 5) for qPCR experiment, followed by blasting of these gene sequences to NCBI database. This study utilized around 1 μg total RNA in every reaction. For removing gDNA, we cultured sample for a 2-min period under 42°C and for another 5-min period under 60°C. Reaction buffer (10 μl) in step 1 (20-μl system) was utilized in every reverse transcription. After mixing, the mixture was subject to 30-min incubation under 50°C and 5-min heat shock under 85°C. The PCR conditions were shown below, 30-s under 95°C; 5-s under 95°C and 55-s under 60°C for altogether 40 cycles. Meanwhile, this work utilized gene-specific primers (F/R) of 20 candidate DEGs and the kenaf internal control gene GhACTIN (GenBank accession no. AY305733) (Lu et al., 2018), with action being the control. Further, 2^–ΔΔ*Ct*^ approach was utilized for determining 20 gene levels. qRT–PCR procedure was conducted in triplicate.

### Parallel Reaction Monitoring Analysis

For verifying the TMT-measured protein levels, a PRM experiment was adopted for further quantifying the expression level of the selected proteins. For every sample, we added the PRTC stable isotope peptide as control. After protein extraction, sample (200 μg) was subject to trypsin hydrolysis. Meanwhile, the C18 cartridge was utilized to desalt peptides; 40 μl of freeze-dried peptide was added to 0.1% formic acid solution, followed by peptide quantification. Peptides of the same mass were mixed and separated by Easy nLC-1200 system (Thermo Fisher Scientific, MA, United States). Meanwhile, this work utilized acetonitrile (ACN) within 40 min with a 1-h liquid chromatographic gradient of 5–30%, and PRM mass spectrometry was carried out using the Q Exactive HF mass spectrometer (Thermo Scientific) in the positive-ion mode, with a full MS1 scanning resolution ratio of 60,000 (200 m/z). This work also set the target values of maximal ion injection time and automatic gain control (AGC) at 200 ms and 3e6, separately; after full MS scanning, 25 PRMs (MS2 scans) were performed, at the resolution and maximal injection time of 120 ms and 30,000 (m/z 200), and agc3e6. In addition, a second window was utilized to isolate target peptides; whereas the normalized collision energy (Grzeszczuk et al., 2018) was adopted for ion activation/dissociation within the high energy dissociation (HCD) collision pool. The 20 target proteins quantified by PRM were imported into Skyline software^6^, the peptide settings were selected, the background proteome database file was added, the protein quantitative polypeptide was selected according to the ion signal in the spectral library, the list of related peptides with retention time was derived from Skyline, and the quantitative results of each peptide of target protein were manually checked. Finally, each sample took 1 μg with 20 fmol heavy isotope-labeled peptide section PRTC as the internal label, and the target peptides and proteins were quantitatively analyzed by the same chromatographic conditions and mass spectrometry methods as the previous PRM method. The signal strength of the single peptide sequence of each significantly changed protein was measured for all samples relative to control (Gillet et al., 2012).

## Results

### Analysis of the Crude Protein Content in Kenaf Feed

**FIGURE 1:**
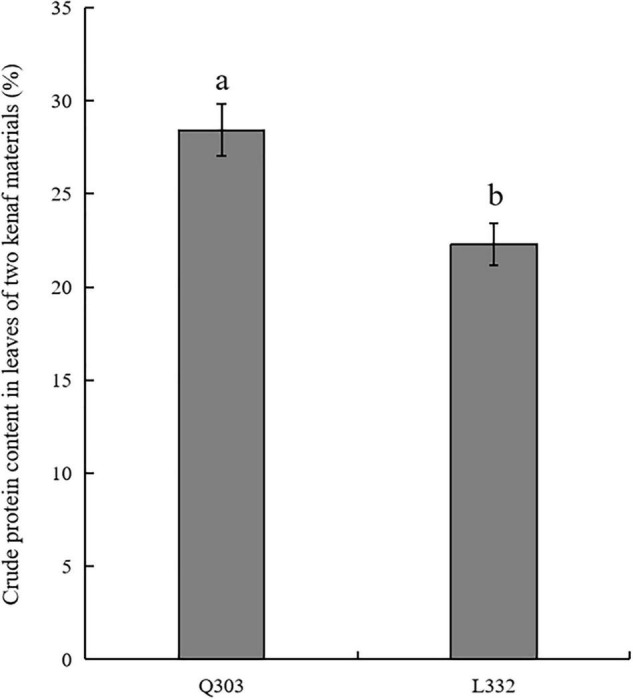
Crude protein content in leaves from two kenaf materials. This assay was carried out in 3 biological replicates, with 3 technical replicates being set each. Student’s *t*-test was adopted for data analysis (*p* < 0.05). Error bars = standard error of the mean (SEM). Different letters indicate significant difference at *p* < 0.05.

Kenaf leaves are applied as livestock feed because of their high protein concentration and large biomass. The crude protein in leaves of two kenaf materials were determined. As a result, mature “Q303” leaves had markedly increased protein levels compared with mature “L332” (Figure 1). The average percentage of crude protein content in leaves of kenaf material “Q303” reached 28.43%. Compared with kenaf material “Q303,” the percentage of crude protein content in leaves of material “L332” decreased by 6.13%.

### Transcriptomic Analysis Overview

For summarizing the transcriptomes of the two kenaf materials in the mature period, this work built six cDNA libraries (i.e., L332 and Q303, three repeats). Altogether 49.33 and 47.74 million raw sequences were generated in cleft Q303 and L332 libraries, separately. Later, adaptor sequences, low-quality reads and those with uncertain base ratios >10% were eliminated, leaving 46.32 and 47.78 million clean reads with Q30 base percentages and GC contents of 94.23-94.38 and 47.07-48.00%, respectively (Supplementary Table 1). A total of 222,979 transcripts (range, 201–2,000 bp), with altogether 101,679 unigenes (>200 bp) were discovered (Table 1). The size distributions of unigenes and transcripts are presented in Supplementary Figure 1.

**TABLE 1:** Summary of the proteomic and transcriptomic data within leaves of two kenaf materials.

RNA-seq data		MS data based on transcriptome	
Total number of transcripts	222,979	Match spectra	66,347
Mean length of transcripts (bp)	1,216	Identified peptides	32,558
Total number of unigenes	101,679	unique peptides	26,972
Mean length of unigenes (bp)	821	Identified proteins	7,196
N50 length of transcripts (bp)	1,919		
N50 length of unigenes (bp)	1,551		

For determining those identified transcripts’ candidate functions, this study utilized Basic Local Alignment Search Tool (BLAST) to annotate unigenes on the basis of 5 databases, like National Center for Biotechnology Information non-redundant protein sequences (NR) (60,171, 59.18% of all the identified unigenes), SwissProt (40,606, 39.94%), Protein families (Pfam) (31,431, 30.91%), Gene Ontology (GO) (34,448, 33.88%), and KEGG (10,456, 10.28%) databases. Based on the above results, NR database had the most functional annotations, which suggested that all the 61141 unigenes corresponded to sequences from one or more public databases. Finally, there were 6879 functionally annotated unigenes from different databases (Supplementary Table 2).

Of those, 3,778 DEGs were found by *p* < 0.05 and log2-FC > 1 thresholds in leaves of two kenaf materials. Q303 group showed 1,350 and 2,428 genes with up-regulation and down-regulation, respectively, relative to L332 group. Supplementary Figure 2A displays a volcano plot of the above findings. Table 2 summarized data regarding the identified DEGs. Many of the above genes obtained from annotation had not been characterized before.

**TABLE 2:** Proteins and transcripts measured based on TMT and RNA sequence data.

	Transcriptome	Protein
Unique proteins/genes detected	101,679	7,196
Significantly DEGs/DAPs	3,778	1,320
Up-regulated	1,350	636
Down-regulated	2,428	684
Co-regulated DEGs-DAPs	134	134
Co-regulated DEGs-DAPs with the same trends	122	122
Co-regulated DEGs-DAPs with the opposite trends	12	12

### Functional Classification of the Identified Differentially Expressed Genes

**FIGURE 2:**
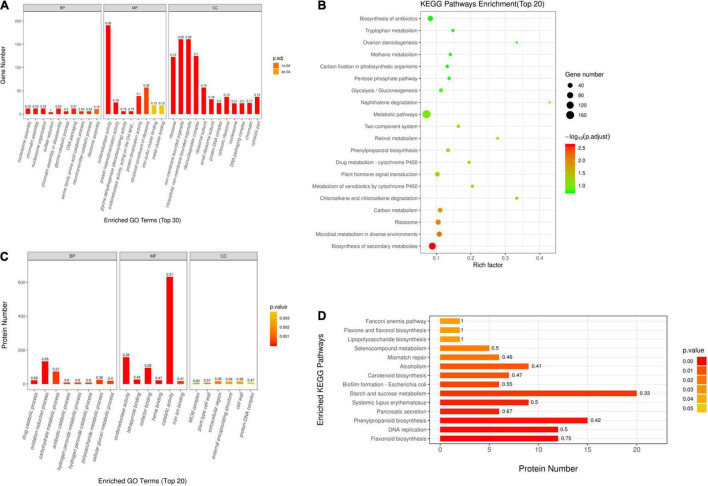
Gene Ontology (GO) and Kyoto Encyclopedia of Genes and Genomes (KEGG) analyses on DAPs and DEGs. **(A)** GO annotations for DEGs within Q303/L332. Those 30 most significant BP, MF, and CC terms are presented in horizontal axis, whereas gene numbers of GO categories are displayed in vertical axis. **(B)** GO annotations for DAPs within Q303/L332. Those 20 most significant BP, MF, and CC terms are presented in horizontal axis, whereas protein numbers of GO categories are displayed in vertical axis. In every histogram, color and height stand for *p*-value and protein/gene count, separately. The histogram label stands for rich factor and it indicates the ratio of DEG/DAP number to overall gene/protein number of GO terms**. (C)** KEGG analysis on DEGs within Q303/L332. **(D)** KEGG pathways enrichment of DAPs in Q303/L332. Enrichment factors of diverse KEGG pathways **(C)** and protein counts in diverse terms **(D)** are displayed in horizontal axis. Those most significant KEGG pathways are presented in vertical axis. The circle color and size stand for *p*-value and gene count of each KEGG term, separately. The histogram color and length represent *p*-value and protein count of each KEGG term. The histogram label stands for rich factor, and it indicates the ratio of DEG/DAP number to overall gene/protein number of KEGG categories.

For better understanding DEGs’ function, this work carried out bioinformatics analysis through hierarchical clustering and GO analysis. As revealed by GO functional annotation, many DEGs were enriched into serine family amino acid catabolic process and glycine catabolic process with regard to BP terms; oxidoreductase activity and glycine dehydrogenase (decarboxylating) activity with regard to MF terms; and ribosome and intracellular non-membrane-bounded organelle with regard to CC terms (Figure 2A).

**FIGURE 3:**
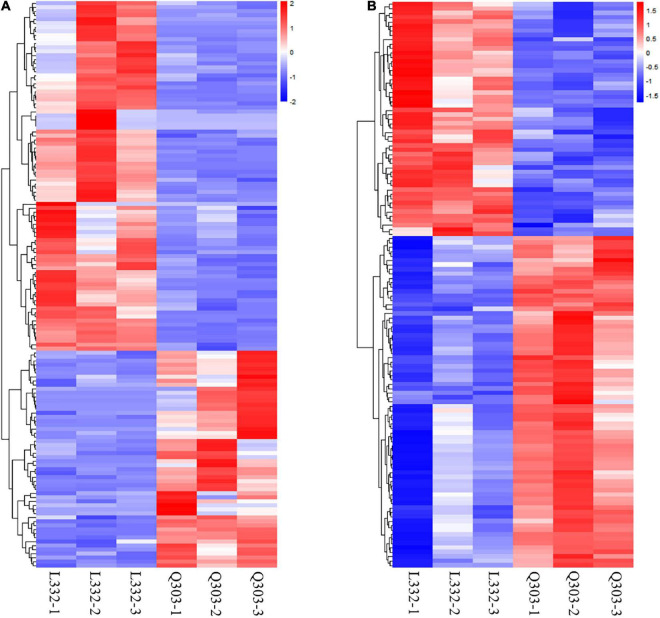
Heatmap showing DAPs and DEGs through proteomic and transcriptomic analyses, and they were related to the protein synthetic metabolic processes. Red, blue, and white stand for markedly up-regulated, down-regulated and non-significant proteins, separately. **(A)** Heatmap of protein synthesis-related gene expression. Amino acid biosynthesis and metabolism, energy metabolism, carbohydrate metabolism, genetic information processing, and carbon metabolism were clustered closely. **(B)** Heatmap of protein synthesis-related protein expression. Including biosynthesis of amino acids, the nitrogen metabolism pathway, starch and sucrose metabolism, carbon metabolism, glycine/serine/threonine metabolism, aminoacyl-tRNA biosynthesis, and the protein synthesis-related proteins were clustered closely.

Additionally, this work conducted KEGG analysis for evaluating DEGs. As a result, most DEGs were associated with secondary metabolite biosynthesis and antibiotic metabolic pathway biosynthesis in the leaves of the two kenaf materials (Figure 2C). Besides, this study conducted hierarchical clustering for improving the understanding on alterations of protein synthesis-associated DEG levels (Figure 3A). In all, 141 protein synthesis-related pathways, including amino acid biosynthesis and metabolism, energy metabolism, carbohydrate metabolism, genetic information processing, and carbon metabolism, were clustered closely.

### Quantitative Proteome Analysis

For exploring the mechanism underlying high protein content in kenaf leaves, this work carried out quantitative proteomics analysis by LC-MS/MS analysis and TMT platform in mature stage, for the sake of complementing transcriptomic analysis. Proteomic analysis identified altogether 66,347 spectra, 26,972 unique peptides and 32,558 identified peptides, among which, we discovered 7,196 proteins (Table 1 and Supplementary Table 3). As for distribution of protein mass, proteins whose molecular weights (MWs) were >9 kDa showed a broad range as well as favorable coverage, and the maximal distribution area reached 10–40 kDa (Supplementary Figure 3A). According to protein peptide quantification, protein number declined as matching peptide increased (Supplementary Figure 3B).

Of the above proteins, we discovered 1,320 differentially abundant protein (DAPs) upon the upregulated and downregulated thresholds being FC > 1.2 as well as *p* < 0.05, separately, including 636 with higher abundance levels, whereas 684 with lower abundance levels between Q303/L332 groups, as displayed by the volcano plot Supplementary Figure 2B.

### Functional Classification of the Identified Differentially Abundant Proteins

#### Gene Ontology Analysis

This work also conducted bioinformatics analysis on those identified DAPs according to hierarchical clustering and functional classifications of proteins. According to GO annotation, many DAPs were enriched into oxidation-reduction process and carbohydrate metabolic process with regard to BP terms. As for MF terms, DAPs were mostly enriched into oxidoreductase activity and catalytic activity. In terms of CC category, DAPs were enriched into plant-type cell wall and protein-DNA complex (Figure 2B).

#### Kyoto Encyclopedia of Genes and Genomes Analysis

This work also performed KEGG analysis for evaluating DAPs. Many DAPs were associated with phenylpropanoid biosynthesis, DNA replication, and starch/sucrose metabolism pathways (Figure 2D).

#### Hierarchical Clustering Analysis

This work also conducted hierarchical clustering for better exploring alterations of protein synthesis-associated DAPs levels. Altogether 128 DAPs related to protein synthesis, which included biosynthesis of amino acids, the nitrogen metabolism pathway, carbon metabolism, starch/sucrose metabolism, glycine/serine/threonine metabolism, protein synthesis-related proteins, and aminoacyl-tRNA biosynthesis, were clustered closely (Figure 3B).

#### PPI Network Analysis

**FIGURE 4:**
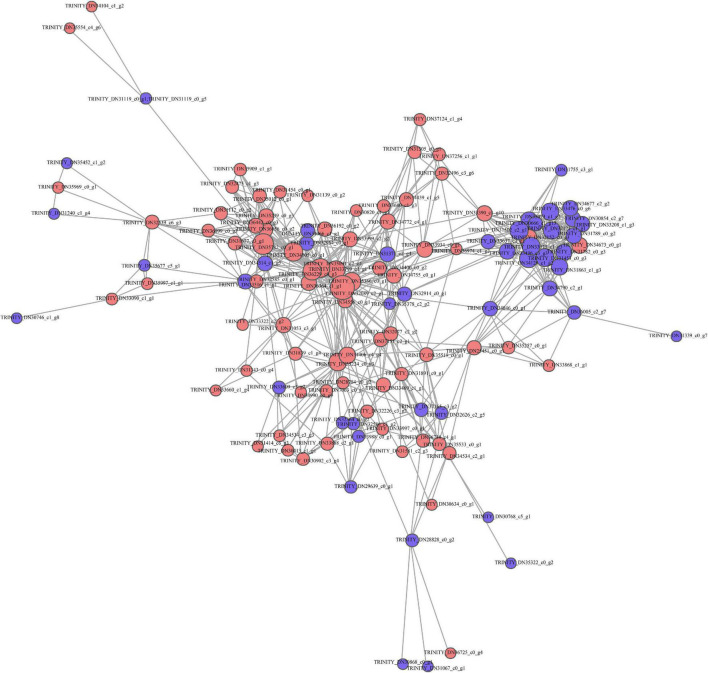
Functional annotation of DAPs based on STRING 9.0. Red and blue stand for markedly up-regulated and down-regulated proteins, separately.

This work established a PPI network based on STRING database to predict the biological function of protein synthesis in kenaf leaves. Most differentially expressed proteins are associated with functional interactions. A total of 128 DAP-related protein syntheses in kenaf leaves, including 75 upregulated and 53 downregulated DAPs, were incorporated in interaction network (Figure 4).

### Comparison Gene Levels and Protein Levels

**FIGURE 5:**
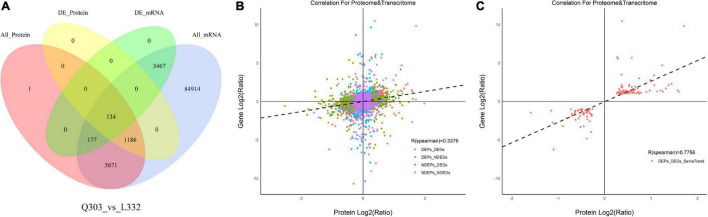
Associations of protein and mRNA levels. **(A)** Venn diagram showing proteome (pink)-, transcriptome (blue)-quantified proteins, DAPs (yellow), and DEGs (green) within Q303/L332. **(B)** Scatterplot of the relationship between genes identified in proteomic and transcriptomic analyses of Q303/L332. **(C)** Scatterplot showing the correlation coefficients of DEGs with DEPs (the same trend) in Q303/L332.

A higher number of DEGs (1,350 with up-regulation and 2,428 with down-regulation) was obtained than DAPs (636 with up-regulation and 684 with down-regulation). For evaluating the relation of proteomic and transcriptomic alterations during protein synthesis, correlation analysis was performed using the quantification of DAPs and DEGs. As a result, 134 DAPs and their corresponding DEGs were identified. Of these, 122 DAP abundances increased and 12 DAP abundances decreased (Figure 5A). The results showed that there were more DEGs than DAPs, and there were significant differences in transcription level and protein abundance.

Moreover, Pearson’s correlation test suggested that FCs of DAPs showed moderate correlation with specific DEGs (*r* = 0.3379, *p* < 0.01), which indicated that transcript contents were more closely related to protein levels (Figure 5B). Meanwhile, FCs in DAPs exhibited positive relation to DEGs (*r* = 0.7756, *p* < 0.01) (Figure 5C).

### Identification of Differentially Expressed Genes and Differentially Abundant Protein Related to Candidate Pathways

For better understanding those co-expressed DEG-DAP genes for their biological roles, this work performed enrichment analysis on the basis of GO as well as KEGG analysis. Consequently, the markedly enriched BP terms were cellular processes and metabolic processes, whereas cellular parts and binding and catalytic activities were the obviously enriched CC and MF terms, separately (Supplementary Figure 4A). According to the association analysis, 134 DAPs were enriched in 160 metabolic pathways, 10 significantly enriched pathways in the transcriptome, and 20 significantly enriched pathways in the proteome. There were fifty-seven common pathways between proteomic and transcriptomic data. The comparison demonstrated 2 common pathways, namely, phenylpropanoid biosynthesis, and starch/sucrose metabolism pathways, in proteomic and transcriptomic data (Supplementary Figure 4B). Therefore, these shared metabolic pathways might exert possible functions in the protein synthesis of kenaf leaves.

### Validation of Quantitative Real-Time PCR Gene Expression With RNA-Seq Data

**FIGURE 6:**
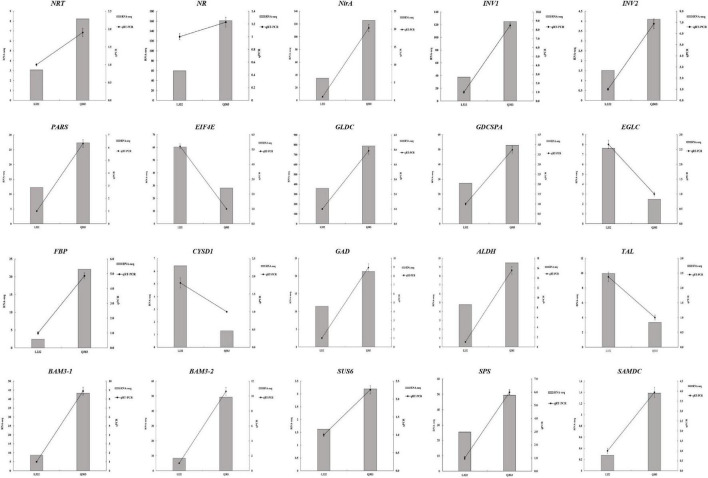
qPCR analysis conducted to validate the chosen gene expression. RNA-seq and qPCR (left and right *y*-axes, separately) were conducted to reveal gene expression. RNA-seq detected gene levels were presented in histograms. qPCR was conducted to validate gene levels, as shown in the line graph.

For validating whether our TMT and RNA-Seq data were reliable, this work conducted correlation and expression analyses by qPCR, and later acquired fragments per kilobase per million reads mapped (FPKM) values based on proteomic and transcriptomic data. Those 20 most significant DEGs were enriched into starch and sucrose metabolism, carbon fixation in photosynthetic organisms, glutathione metabolism, aminoacyl-tRNA biosynthesis, nitrogen metabolism, glycine/serine/threonine metabolism, and other protein synthesis of kenaf leaves were tested by qPCR experiments, and the results are shown in Figure 6. Specifically, most of these DEGs were significantly upregulated, including the starch and sucrose metabolism pathway related genes-sucrose-phosphate synthase (*SPS*), beta-fructofuranosidase (*INV1* and *INV2*), beta-amylase 3 (*BAM3-1* and *BAM3-2*) and sucrose synthase 6 (*SUS6*); glycolysis/gluconeogenesis metabolism related gene-aldehyde dehydrogenase (*ALDH*); carbon fixation in photosynthetic organisms related gene-fructose 1,6-bisphosphate 1-phosphatase activity (*FBP*); nitrogen metabolism related genes-nitrate reductase (*NR*), nitrate transporter (*NRT*), nitrite reductase (*NirA*); serine and threonine metabolism related gene-glycine dehydrogenase (*GLDC*),Glycine dehydrogenase (decarboxylating) A (*GDCSPA*) and aminoacyl-tRNA biosynthesis related gene-proline-tRNA ligase (*PARS*); and cysteine/methionine metabolism associated gene-*S*-adenosylmethionine decarboxylase (*SAMDC*), alanine/aspartate/glutamate metabolism associated gene-glutamate decarboxylase (*GAD*). Translation initiation factor 4E (*EIF4E*), transaldolase-like (*TAL*), glucan endo-1,3-beta-glucosidase (*EGLC*), and L-3-cyanoalanine synthase D-1 (*CYSD1*) were downregulated. Additionally, 14 potential genes, like *FBP* (*r* = 0.9349, *p* < 0.01), *SPS* (*r* = 0.8301, *p* < 0.05), *INV1* (*r* = 0.9106, *p* < 0.05), *INV2* (*r* = 0.7154, *p* < 0.05), *NRT* (*r* = 0.6840, *p* < 0.05), *NR* (*r* = 0.6553, *p* < 0.05), *NirA* (*r* = 0.8855, *p* < 0.05), *PARS* (*r* = 0.8863, *p* < 0.01), *GLDC* (*r* = 0.8451, *p* < 0.01), *GDCSPA* (*r* = 0.7583, *p* < 0.01), *SAMDC* (*r* = 0.7693, *p* < 0.05), *ALDH* (*r* = 0.7815, *p* < 0.05), *EIF4E* (*r* = 0.8543, *p* < 0.01), and *CYSD1* (*r* = 0.7442, *p* < 0.05), were strongly related to RNA-Seq results, whereas 6 genes showed low relation to related protein levels (Figure 6 and Table 3). Generally speaking, qPCR analysis verified gene expression profiles acquired based on proteomic and transcriptomic data, indicating the reliability of our observations.

**TABLE 3:** Association of qPCR with FPKM values for those chosen genes.

Accession	Gene description	Gene name	Pearson correlation efficient	*P*-value	Numbers
TRINITY_DN36229_c0_g3	Fructose 1,6-bisphosphate 1-phosphatase activity	*FBP*	0.9349	0.0007	12
TRINITY_DN31677_c3_g1	Sucrose-phosphate synthase	*SPS*	0.8301	0.0392	12
TRINITY_DN32539_c6_g3	Beta-fructofuranosidase, INV1	*INV1*	0.9106	0.0248	12
TRINITY_DN30709_c0_g1	Beta-fructofuranosidase, INV2	*INV2*	0.7154	0.0306	12
TRINITY_DN36622_c1_g1	Nitrate transporter	*NRT*	0.6840	0.0482	12
TRINITY_DN35530_c0_g1	Nitrate reductase	*NR*	0.6553	0.0330	12
TRINITY_DN32077_c2_g2	Nitrite reductase	*NirA*	0.8855	0.0344	12
TRINITY_DN33868_c1_g1	Proline–tRNA ligase	*PARS*	0.8863	0.0018	12
TRINITY_DN37151_c2_g1	Glycine dehydrogenase	*GLDC*	0.8451	0.0002	12
TRINITY_DN34120_c0_g3	Beta-amylase 3-1	*BAM3-1*	0.4584	0.1844	12
TRINITY_DN34120_c0_g1	Beta-amylase 3-2	*BAM3-2*	0.6295	0.1017	12
TRINITY_DN37151_c2_g4	Glycine dehydrogenase (decarboxylating) A	*GDCSPA*	0.7583	0.0004	12
TRINITY_DN33517_c1_g4	*S*-adenosylmethionine decarboxylase	*SAMDC*	0.7693	0.0060	12
TRINITY_DN30966_c3_g1	Aldehyde dehydrogenase	*ALDH*	0.7815	0.0186	12
TRINITY_DN35582_c0_g6	Glutamate decarboxylase	*GAD*	0.5173	0.7802	12
TRINITY_DN32721_c0_g1	Sucrose synthase 6	*SUS6*	0.3489	0.7694	12
TRINITY_DN31962_c2_g2	Translation initiation factor 4E	*EIF4E*	0.8543	0.0003	12
TRINITY_DN32625_c1_g1	L-3-cyanoalanine synthase D-1	*CYSD1*	0.7442	0.0101	12
TRINITY_DN31110_c0_g7	Transaldolase-like	*TAL*	0.3624	0.1873	12
TRINITY_DN31240_c1_g4	Glucan endo-1,3-beta-glucosidase	*EGLC*	0.3975	0.1172	12

**FIGURE 7:**
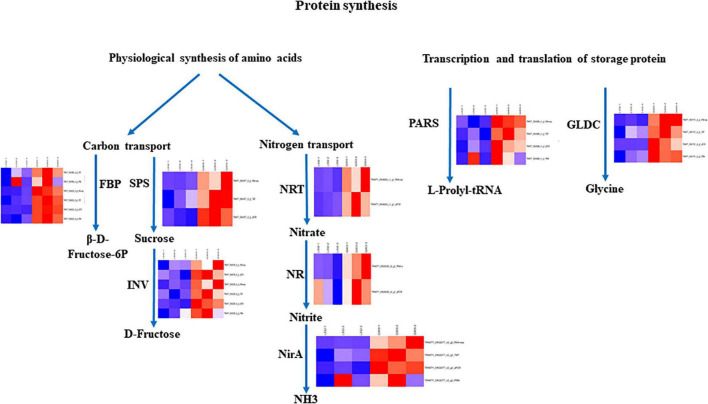
Certain biological pathways related to protein synthesis in kenaf leaves. TRINITY_DN33999_c2_g2 (ALD); TRINITY_DN36622_c1_g1 (NRT), TRINITY_DN35530_c0_g1 (NR); TRINITY_DN30709_c0_g1 (INV2); TRINITY_DN31677_c3_g1 (SPS); TRINITY_DN36229_c0_g3 (FBP); TRINITY_DN32539_c6_g3 (INV1); TRINITY_DN32077_c2_g2 (NirA); TRINITY_DN33868_c1_g1 (PARS); TRINITY_DN37151_c2_g1 (GLDC). Red, blue, and white stand for markedly up-regulated, down-regulated and non-significant proteins, separately.

Moreover, to further validate the proteomics data, we quantified the protein abundance of 40 randomly selected genes by PRM and analyzed the protein abundance of 20 specific genes associated with protein synthesis, 19 of which were successfully quantified. Fifteen of the 19 (78.9%) proteins exhibited close abundance trends in PRM compared with TMT analysis, including ferredoxin-nitrite reductase (NirA), glutathione *S*-transferase DHAR2 (DHAR2), prolyl-tRNA synthetase (PARS), 3-phosphoshikimate 1-carboxy vinyl transferase (EPSPS), fructose-bisphosphate aldolase (ALD), peroxidase (PRX), ferredoxin-NADP+ reductase (petH), catalase isozyme 2 (CAT2), divinyl chlorophyllide an 8-vinyl-reductase (DVR), 1,4-alpha-glucan-branching enzyme 1 (GBE1), glycine dehydrogenase (GLDC), beta-fructofuranosidase INV1 (INV1), fructose 1,6-bisphosphate 1-phosphatase activity (FBP), cysteine synthase (Csase), and glycine-tRNA ligase (GlyRS). Additionally, four genes [glycerate dehydrogenase (HPR), glyceraldehyde-3-phosphate dehydrogenase GAPA2 (GAPA2), fructokinase-2 (FRK2), glutamine synthetase cytosolic isozyme 2 (GS1-2)] exhibited different levels relative to TMT-measured protein levels (Table 4). Thus, the results showed that PRM results were well correlated with proteomics analysis results, besides, some genes related to protein synthesis-associated metabolic pathways were consistently up-regulated and down-regulated within proteome and transcriptome (Figure 7).

**TABLE 4:** Comparison of PRM and TMT quantification results.

Accession	Protein description	Gene name	Q303/L332 ratio	
			PRM	TMT
TRINITY_DN32077 _c2_g2	Ferredoxin-nitrite reductase	NirA	1.253	1.440
TRINITY_DN37151 _c2_g1	Glycine dehydrogenase	GLDC	1.4282	1.529
TRINITY_DN32539 _c6_g3	Beta-fructofuranosidase INV1	INV1	1.176	1.286
TRINITY_DN36229 _c0_g3	Fructose 1,6-bisphosphate 1-phosphatase activity	FBP	1.969	2.714
TRINITY_DN33868 _c1_g1	Prolyl-tRNA synthetase	PARS	1.843	1.486
TRINITY_DN33609 _c3_g2	Glutathione *S*-transferase DHAR2	DHAR2	0.3936	0.7541
TRINITY_DN33988 _c0_g1	3-phosphoshikimate 1-carboxyvinyltransferase	EPSPS	0.9995	0.7456
TRINITY_DN33999 _c2_g2	Fructose-bisphosphate aldolase	ALD	1.718	1.676
TRINITY_DN34106 _c0_g1	Peroxidase	PRX	2.114	2.380
TRINITY_DN34400 _c0_g2	Ferredoxin–NADP+ reductase	petH	1.028	1.387
TRINITY_DN34755 _c0_g1	Glycerate dehydrogenase HPR	HPR	0.3105	1.408
TRINITY_DN35224 _c0_g3	Catalase isozyme 2	CAT2	4.766	1.442
TRINITY_DN35677 _c6_g1	Divinyl chlorophyllide a 8-vinyl-reductase	DVR	1.175	1.392
TRINITY_DN35909 _c1_g1	1,4-Alpha-glucan-branching enzyme 1	GBE1	4.591	1.381
TRINITY_DN33469 _c1_g1	Glutamine synthetase cytosolic isozyme 2	GS1-2	0.5675	1.219
TRINITY_DN33516 _c1_g1	Fructokinase-2	FRK2	1.401	0.6498
TRINITY_DN35366 _c0_g1	Glyceraldehyde-3-phosphate dehydrogenase GAPA2	GAPA2	0.7153	1.375
TRINITY_DN31891 _c0_g1	Cysteine synthase	CSase	1.413	1.257
TRINITY_DN25451 _c0_g1	Glycine-tRNA ligase	GlyRS	1.015	1.283

## Discussion

Kenaf is a new plant protein source, but there are few reports on the mechanism of kenaf leaf protein synthesis. In the current work, by determining the crude protein content in the leaves of two kenaf materials, it was determined that the protein content of the kenaf material “Q303” leaves was higher; combined with multiomics data, kenaf samples were systematically studied to obtain mRNA-protein correlations; then, the genes/proteins related to protein synthesis in kenaf leaves were obtained.

### General Features of the Transcriptomes and Proteomes of Different Kenaf Leaves

The high protein content in leaves is the key factor affecting kenaf’s multi-functional breeding. Illustrating the molecular mechanism of protein synthesis in leaves is helpful for the development of forage products using kenaf leaves. However, the underlying mechanism of kenaf protein synthesis is greatly unknown. According to TMT and RNA-seq data, the current work explored the different proteomic and transcriptomic data of two kenaf materials at the same stage. Since the transcriptome database is applied in identifying proteins, transcriptomic data assembly and sequencing quality is important for the following study. Totally, this work assembled 101,679 unigenes (>200 bp) into kenaf transcriptome, and the total was much higher than that formerly reported for this species as well as other Malvaceae Juss. plants, for example, [55,623 unigenes by Tang et al. (2021); 71,318 unigenes by Li et al. (2016)], and *Hibiscus rosa-sinensis* (30988 unigenes) (Trivellini et al., 2016). The obtained percentages of the Q20 bases (98.08-98.15%), Q30 bases (94.23-94.38%), with close GC concentrations (47.07-48.00%) to other researches on kenaf transcriptome, e.g., (98.19–98.31%), (94.45-94.71%), and (46.15-46.47%) (Tang et al., 2021), respectively. Li et al. (2016) only studied Q20 bases (97.70-97.77%) and GC contents (45.42-46.05%). In total, 60171 (59.18%) and 40606 (39.94%) of all unigenes detected in the present work matched those in SwissProt and NR databases, separately. The findings supported the results obtained by *Hibiscus* L. (20,547 and 13,184; 66.31 and 42.55%) (Trivellini et al., 2016). Therefore, our results provide a wide range of sequence and unigene resources for *Malvaceae* Juss.

In addition, 66,347 match spectra, 26,972 unique peptides, and 7,196 proteins were detected in accordance with the transcriptome data from kenaf leaves. RNA-seq and protein sequencing methods have found and annotated many genes and proteins, providing the foundation for a more correct and thorough description of molecular processes and analysis of genetic regulation mechanisms and complex physiological processes (Vanessa et al., 2012). The data presented here are sufficient and accurate and will provide useful guidance tools for the study of protein synthesis in kenaf leaves and other mallow plants. In addition, there were a lot of “predicted,” “putative,” or “uncharacterized” transcripts and proteins in the annotation results, suggesting that the nature of the analysis was restricted because of the lack of detailed genomic information (Niu et al., 2020). As a result, the functions of these unknown or unidentified genes and proteins in the protein synthesis of kenaf leaves should be explored in further researches.

### Translational and Post-translational Regulation on Protein Accumulation in Kenaf Leaves

The regulation of storage protein gene expression is complex and includes transcription regulation, processing regulation for transcription products, translation regulation, hormone regulation, and so on (Kroj et al., 2003). As a result, the differences in the expression of genes and proteins in a single period alone cannot fully and correctly reflect the cause of protein accumulation in kenaf leaves. In the current work, the comparative analysis of gene expression and protein levels suggested that there existed more DEGs than DAPs. This may be due to protein post-translational modifications that manage the degradation or secretion of the protein, the technical limitations of MS-based proteomics, and the availability of a few samples. These reasons limit the ability to identify proteins (Zhou et al., 2015). Investigating the association between proteins and the network formed by their interaction is significant to show the roles of proteins (Xing et al., 2016). The PPI analysis showed that the interaction between proteins was obvious, and there was a complex network between interacting proteins. The interactions between proteins constitute a major part of the cellular biochemical reaction network, which is of great significance in regulating cells and their signals (Liu et al., 2021). In addition, correlation analysis demonstrated that there existed a weak correlation between the proteome and transcriptome, suggesting that there existed low correlation between transcription level and protein abundance. The findings were similar to those found in former studies, indicating that post-transcriptional and post-translational regulation, reversible phosphorylation, cell splicing events and translation efficiency exert a vital function in the regulation of protein accumulation in leaves (Chen et al., 2019). Therefore, gene translation and post-translational modification could be important methods of protein accumulation in leaves.

### Potential Regulators and Metabolic Pathways During the Process of Protein Accumulation in Leaves

The GO and KEGG functional enrichment provides the definition and description of gene and protein functions and integrates genomic, chemical and systemic functional information. GO term analysis showed that in addition to the co-enrichment of glycine dehydrogenase (decarboxylation) activity and serine family amino acid catabolic processes related to amino acid metabolism, oxidoreductase activity was also co-enriched. Similar to former researches, the target genes can be classified into different categories based on their roles, such as amino acid anabolism and peroxidase activity (Skadsen and Cherry, 1983). Peroxidase participates in the photorespiration of plant cells and redox equilibrium reactions (Jin et al., 2013). Glycine is mainly used as a glycine residue in leaf protein synthesis (Guo et al., 2021). These modifications improved the efficiency of photosynthesis and provided raw materials for protein synthesis. KEGG analysis showed that starch and sucrose metabolism is a shared pathway of DEGs and DAPs and other pathways involved in carbon metabolism, which provide basic substances for protein synthesis. Starch and sucrose metabolism can generate a vital function in the process of protein accumulation in kenaf leaves. In addition to the 21 amino acid synthesis pathways, other metabolic pathways are indirectly involved in amino acid synthesis (Iqbal et al., 2020). These processes include gluconeogenesis, the pentose phosphate pathway, the citrate cycle, carbon fixation in photosynthetic organisms as well as pyruvate metabolism. These metabolic pathways are closely related to the synthesis of amino acids and provide raw materials for protein synthesis. Furthermore, the findings showed that the proteomic data and transcriptome data were complementary and that the proteome could verify the transcriptome data; besides, genes exert the same role at the transcriptome and proteome levels (Muers, 2011). Besides, the functional classification of the transcriptome and proteome is conducive to improving the comprehension of protein synthesis physiology and molecular biology.

### Key Candidate Differentially Expressed Genes and Differentially Abundant Proteins Involved in Leaf Protein Synthesis

Protein synthesis is a complex physiological, biochemical, and molecular biological process. It is not directly determined by a single gene but is jointly controlled by a lot of genes with different functions (Skadsen and Cherry, 1983). Fructose-1,6-bisphosphate aldolase (ALD) is a critical enzyme for the conversion from 3C to 6C compounds after CO_2_ fixation. The upregulated ALD catalyzes the alcohol aldehyde condensation of dihydroxyacetone phosphate (DHAP) and glyceraldehyde 3-phosphate (GAP) to produce more fructose-1,6-diphosphate (FDP). Fructose-1,6-bisphosphatase (FBP) catalyzes fructose-1,6-diphosphate (FDP) and water with the purpose of generating fructose-6-phosphate and inorganic phosphorus, providing raw material for protein synthesis and has been verified in other crops (Li et al., 2022). Upregulated sucrose phosphate synthase (SPS) catalyzes UDP glucose and fructose 6-phosphate in order to yield more sucrose 6-phosphate (S6P), and sucrose 6-phosphate is hydrolyzed to produce sucrose. The main enzymes of sucrose catabolism are sucrose invertase (INV) and sucrose synthase (SUS), which degrade sucrose to glucose and fructose (Duan et al., 2021). The sucrose synthesized by photosynthesis is converted into reducing sugar by INV to supply the growth of young tissues and provide raw materials for protein synthesis (Deng et al., 2019). Nitrate transporters (NRTs) are a key factor in plant perception, absorption, and transport of nitrate (Zhang et al., 2021). The first step of nitrate degradation occurs in the cytoplasm. Nitrate is lowered to nitrite by nitrate reductase (NR). Nitrite enters chloroplasts or plastids and is degraded to ammonium by nitrite reductase (NirA) to provide nitrogen for protein synthesis (Eady et al., 2016). Aminoacylation of tRNA is the first step in protein synthesis. In this process, aminoacyl tRNA synthetases (aaRSs) connect specific amino acids to homologous tRNA to complete protein synthesis (Smirnova et al., 2012). Some examples include Val tRNA synthetase and threonyl tRNA synthetases (Wang et al., 2016; Zhang et al., 2017). Amino acids play a vital function in plant central metabolism. Amino acids can also play the role of intermediates of final metabolites in some metabolic pathways and be engaged in regulating various metabolic pathways and other physiological and biochemical pathways, thereby influencing protein synthesis. The Aspartic acid (Asp) participates in protein synthesis and provides raw materials for protein synthesis (Yang et al., 2020).

In the current work, 14 DEGs and 15 DAPs which were engaged in protein synthesis presented strong associations with RNA-seq data and protein expression levels, and these correlations are consistent with the results that the metabolic pathway that is directly or indirectly involved in amino acid synthesis may play a key role in protein synthesis (Iqbal et al., 2020). Obviously, the expression levels of four proteins (FBP, NirA, PARS and GLDC) related to carbon fixation in photosynthetic organisms, nitrogen metabolism, aminoacyl-tRNA biosynthesis and glycine, serine and threonine metabolic pathways demonstrated higher protein and gene expression in kenaf leaves with high protein content, indicating FBP, NirA, PARS and GLDC may exert an essential function in protein synthesis of kenaf leaves.

The protein and gene expression levels of ALD, SPS, INV, NR, and NRT in kenaf leaves with a high protein content were higher than those of kenaf leaves with a low protein content. The expression of metabolic proteins related to carbon transport and nitrogen transport increased significantly, indicating that the physiological synthesis of raw amino acids and the transcription and translation of storage protein genes constitute a network in kenaf leaves with high protein content. In future work, transgenic plants are required to overexpress candidate genes to verify the functions of these genes.

## Conclusion

In the present study, two kenaf cultivars with significant differences in protein content were used as materials. The leaf protein content were determined using the Kjeldahl method during the most important kenaf growth period. More importantly, the potential genes/proteins involved in protein synthesis in kenaf leaves were identified by comparative transcriptomic analysis and proteomic analysis. Protein content is regarded as a typical quality trait regulated by multiple genes, and the genetic mechanism are complex and related to multiple pathways. The four co-expressed genes (*FBP*, *NirA*, *PARS*, and *GLDC*) identified in this study were suggested involved in carbon transport metabolism, nitrogen transport metabolism and multiple signaling pathways for the transcription and translation of storage protein genes. The research provides new insight into the process of protein synthesis in kenaf leaves.

## Data Availability Statement

The datasets presented in this study can be found in online repositories. The names of the repository/repositories and accession number(s) can be found in the article/Supplementary Material.

## Author Contributions

ChZ and YD were in charge of study design and manuscript drafting. GZ, JL, AX, LZ, AC, HT, LC, and GP assisted in data analysis. YW, JZ, CuZ, ZB, HL, JW, and DY contributed to obtaining and interpreting data in this study. SH and DL were responsible for manuscript revision. All authors approved the final manuscript for submission.

## Conflict of Interest

The authors declare that the research was conducted in the absence of any commercial or financial relationships that could be construed as a potential conflict of interest.

## Publisher’s Note

All claims expressed in this article are solely those of the authors and do not necessarily represent those of their affiliated organizations, or those of the publisher, the editors and the reviewers. Any product that may be evaluated in this article, or claim that may be made by its manufacturer, is not guaranteed or endorsed by the publisher.

## Footnotes

1
https://github.com/trinityrnaseq/trinityrnaseq/releases

2
http://planttfdb.gao-lab.org/index.php

3
http://string-db.org

4
http://jtreeview.sourceforge.net

5
http://bonsai.hgc.jp/~mdehoon/software/cluster/index.html

6
https://skyline.ms/

## Supplementary Material

Supplementary Figure 1 Size distributions of kenaf’s unigene and assembled transcript sequences.

Supplementary Figure 2 Volcano plot showing kenaf’s proteomic and transcriptomic data. **(A)** Volcano plot showing transcriptomic data of Q303/L332. **(B)** Volcano plot showing proteomic data of Q303/L332. Absolute log2-FC and log10-FC values serve as *x*- and *y*-axes, separately.

Supplementary Figure 3 TMT proteomics-identified peptide counts and molecular weights (MWs) based on database. **(A)** Protein distribution across diverse MWs. **(B)** TMT-detected peptide quantity in proteins.

Supplementary Figure 4 Gene Ontology as well as KEGG analysis of co-expressed proteins and genes within kenaf. **(A)** GO functional annotation of co-expressed proteins and genes within kenaf. **(B)** KEGG analysis on co-expressed proteins and genes within kenaf.

Supplementary Table 1 Kenaf’s sequencing statistics.

Supplementary Table 2 Unigene annotational statistics.

Supplementary Table 3 Overall protein and transcript quantities.

Supplementary Table 4 Differentially abundant proteins (DAPs) and differentially expressed genes (DEGs) discovered.

Supplementary Table 5 Primer sequences utilized in qPCR assay.
