# Supplementary figures and images for: Comparative Transcriptome and Proteome Analysis Provides New Insights Into the Mechanism of Protein Synthesis in Kenaf (Hibiscus cannabinus L.) Leaves
Source: Front Plant Sci. 2022 Jun 21;13:879874. doi: 10.3389/fpls.2022.879874 (PMC9255553; doi:10.3389/fpls.2022.879874)

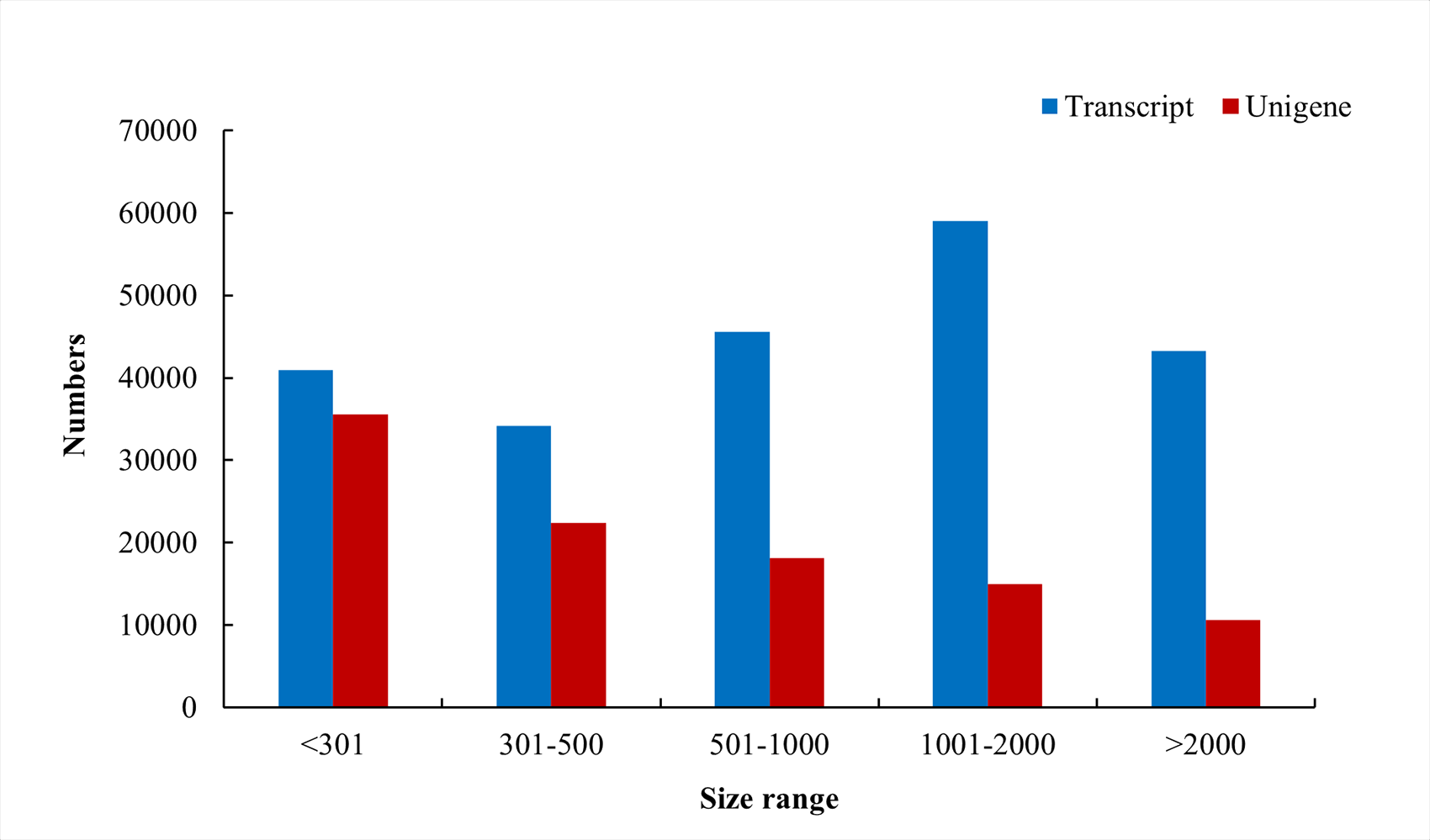

Supplement: Supplementary Figure 1 — Size distributions of kenaf’s unigene and assembled transcript sequences. [file Image_1.TIF]

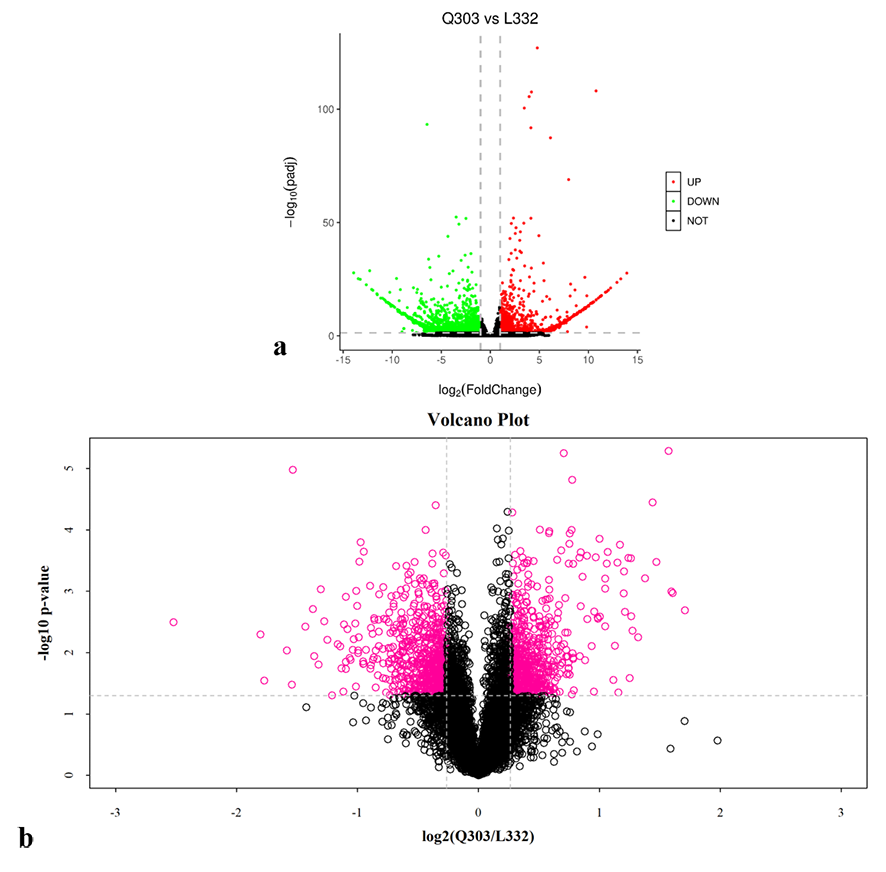

Supplement: Supplementary Figure 2 — Volcano plot showing kenaf’s proteomic and transcriptomic data. (A) Volcano plot showing transcriptomic data of Q303/L332. (B) Volcano plot showing proteomic data of Q303/L332. Absolute log2-FC and log10-FC values serve as x- and y-axes, separately. [file Image_2.TIF]

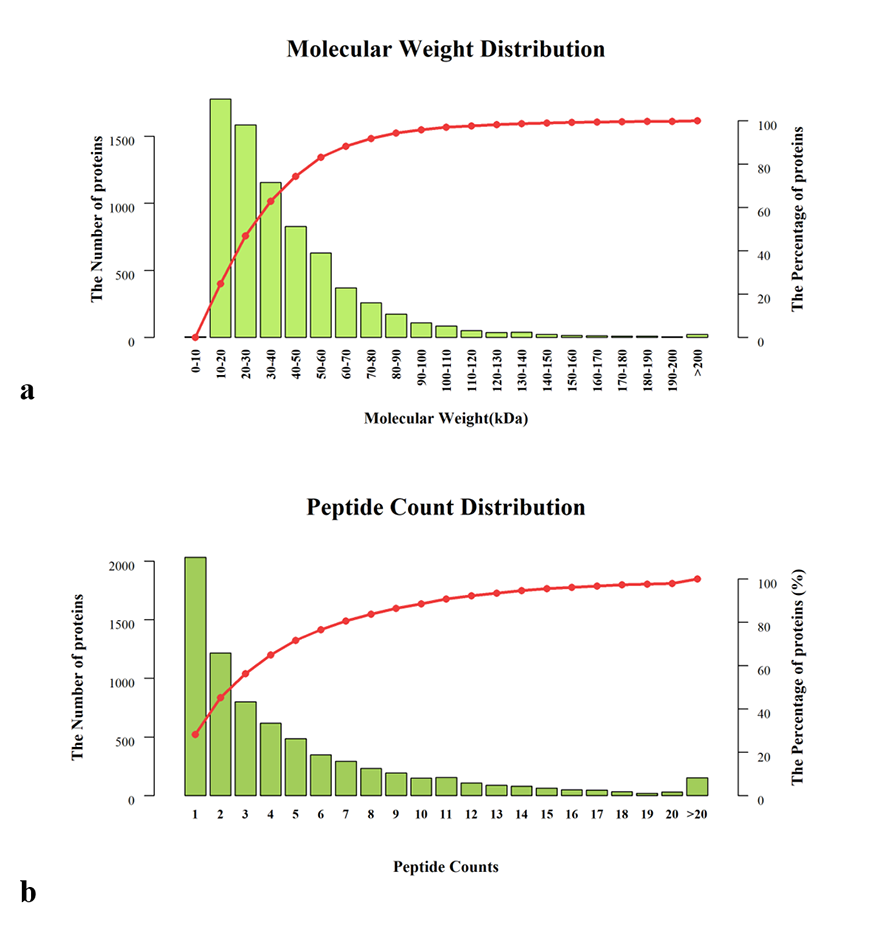

Supplement: Supplementary Figure 3 — TMT proteomics-identified peptide counts and molecular weights (MWs) based on database. (A) Protein distribution across diverse MWs. (B) TMT-detected peptide quantity in proteins. [file Image_3.TIF]

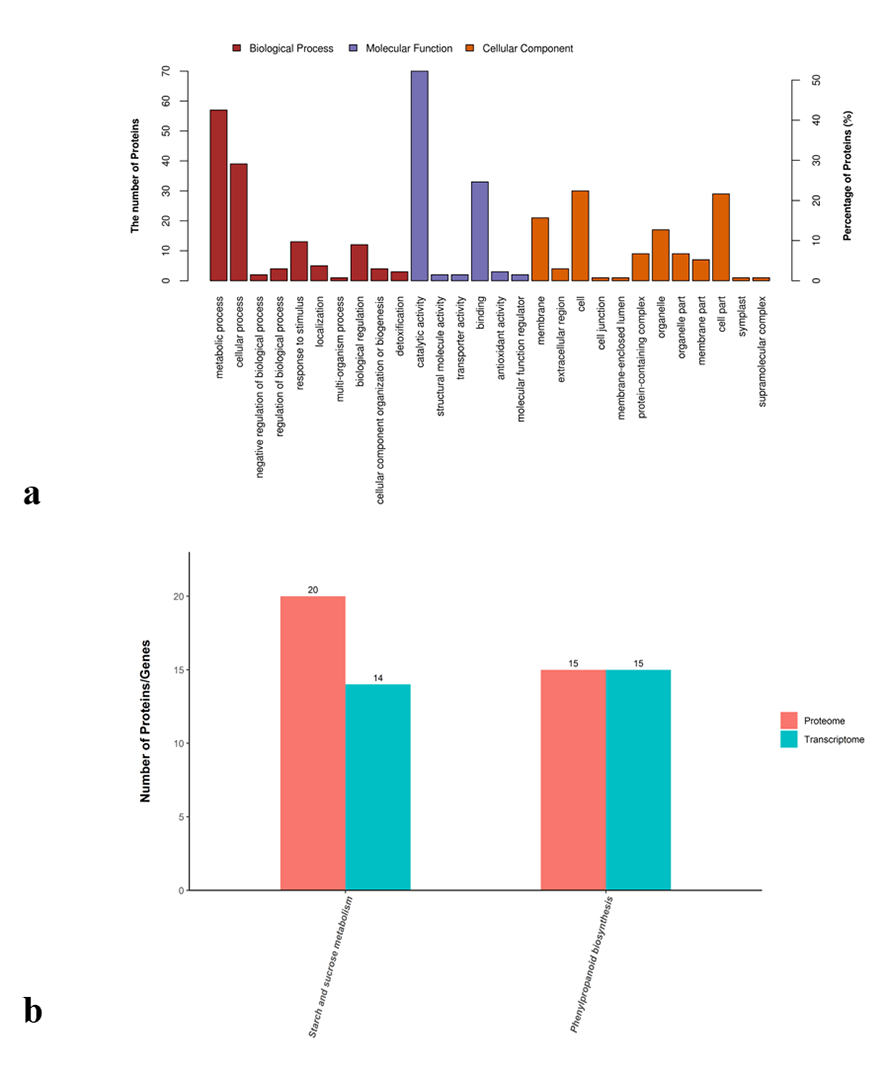

Supplement: Supplementary Figure 4 — Gene Ontology as well as KEGG analysis of co-expressed proteins and genes within kenaf. (A) GO functional annotation of co-expressed proteins and genes within kenaf. (B) KEGG analysis on co-expressed proteins and genes within kenaf. [file Image_4.TIF]
