# Supplementary material for: Comparative Transcriptome and Proteome Analysis Provides New Insights Into the Mechanism of Protein Synthesis in Kenaf (Hibiscus cannabinus L.) Leaves
Source: Front Plant Sci. 2022 Jun 21;13:879874. doi: 10.3389/fpls.2022.879874 (PMC9255553; doi:10.3389/fpls.2022.879874)
Supplement: Supplementary Table 1 — Kenaf’s sequencing statistics. [file Table_1.docx]

**Table S1 | Kenaf’s sequencing statistics**

|  | Sample | Raw | Clean | Clean | Error | Q20 | Q30 | GC |
| --- | --- | --- | --- | --- | --- | --- | --- | --- |
|  |  | reads | reads | Bases (G) | (%) | (%) | (%) | (%) |
|  | L332-1 | 46755526 | 45503140 | 6.26 | 0.02 | 98.15 | 94.37 | 47.07 |
| L332 | L332-2 | 52040856 | 50429054 | 6.93 | 0.02 | 98.13 | 94.38 | 48.00 |
|  | L332-3 | 44429020 | 43037542 | 5.92 | 0.02 | 98.15 | 94.37 | 47.07 |
|  | Mean | 47741801 | 46323245 | 6.37 | 0.02 | 98.14 | 94.37 | 47.38 |
|  | Q303-1 | 48808510 | 47251660 | 6.49 | 0.02 | 98.11 | 94.24 | 47.18 |
| Q303 | Q303-2 | 48887118 | 47231648 | 6.48 | 0.02 | 98.08 | 94.23 | 47.29 |
|  | Q303-3 | 50283676 | 48870302 | 6.72 | 0.02 | 98.10 | 94.28 | 47.29 |
|  | Mean | 49326435 | 47784537 | 6.56 | 0.02 | 98.10 | 94.25 | 47.25 |
