# Supplementary material for: Comparative Transcriptome and Proteome Analysis Provides New Insights Into the Mechanism of Protein Synthesis in Kenaf (Hibiscus cannabinus L.) Leaves
Source: Front Plant Sci. 2022 Jun 21;13:879874. doi: 10.3389/fpls.2022.879874 (PMC9255553; doi:10.3389/fpls.2022.879874)
Supplement: Supplementary Table 2 — Unigene annotational statistics. [file Table_2.docx]

**Table S2 | Unigene annotational statistics**

| Annotated databases | Number of annotated unigenes | | Ratio of annotated unigenes (%) |
| --- | --- | --- | --- |
| NR | | 60171 | 59.18 |
| SwissProt | | 40606 | 39.94 |
| GO | | 34448 | 33.88 |
| PFAM | | 31431 | 30.91 |
| KO | | 10456 | 10.28 |
| Annotated in all Databases | | 6789 | 6.77 |
| Annotated in at least one Database | | 61141 | 60.13 |
| Total Unigenes | | 101679 | 100 |
